# Winter cover crops on processing tomato yield, quality, pest pressure, nitrogen availability, and profit margins

**DOI:** 10.1371/journal.pone.0180500

**Published:** 2017-07-06

**Authors:** Kimberly D. Belfry, Cheryl Trueman, Richard J. Vyn, Steven A. Loewen, Laura L. Van Eerd

**Affiliations:** 1 University of Guelph Ridgetown Campus, Ridgetown, Ontario, Canada; 2 Department of Food, Agricultural and Resource Economics, University of Guelph Ridgetown Campus, Ridgetown, Ontario, Canada; 3 School of Environmental Sciences, University of Guelph Ridgetown Campus, Ridgetown, Ontario, Canada; University of Kentucky, UNITED STATES

## Abstract

Much of cover crop research to date focuses on key indicators of impact without considering the implications over multiple years, in the absence of a systems-based approach. To evaluate the effect of three years of autumn cover crops on subsequent processing tomato (*Solanum lycopersicum* L.) production in 2010 and 2011, a field split-split-plot factorial design trial with effects of cover crop type, urea ammonium nitrate fertilizer rate (0 or 140 kg N ha^-1^ preplant broadcast incorporated) and tomato cultivar (early vs. late) was conducted. The main plot factor, cover crop, included a no cover crop control, oat (*Avena sativa* L.), winter cereal rye (hereafter referred to as rye) (*Secale cereale* L.), oilseed radish (OSR) (*Raphanus sativus* L. var. *oleiferus* Metzg Stokes), and mix of OSR and rye (OSR + rye) treatments. Cover crop biomass of 0.5 to 2.8 and 1.7 to 3.1 Mg ha^-1^ was attained in early Oct. and the following early May, respectively. In general, OSR increased soil mineral N during cover crop growth and into the succeeding summer tomato growing season, while the remaining cover crops did not differ from the no cover crop control. The lack of a cover crop by N rate interaction in soil and plant N analyses at harvest suggests that growers may not need to modify N fertilizer rates to tomatoes based on cover crop type. Processing tomato fruit quality at harvest (rots, insect or disease damage, Agtron colour, pH, or natural tomato soluble solids (NTSS)) was not affected by cover crop type. In both years, marketable yield in the no cover crop treatment was lower or not statistically different than all planted cover crops. Partial profit margins over both years were 1320 $ ha^-1^ higher with OSR and $960 higher with oat compared to the no cover crop control. Thus, results from a systems-based approach suggest that the cover crops tested had no observed negative impact on processing tomato production and have the potential to increase marketable yield and profit margins.

## Introduction

Processing tomato is among Canada’s most lucrative field-grown vegetable crops, at an estimated annual farm gate value over $70 million CAD [1]. With more than half a million tonnes produced per year nationally, up to 90% of Canada’s field tomatoes are grown in Ontario [1]. Processing tomato production in Canada takes place largely in two southwestern Ontario counties, Essex and Chatham-Kent. These regions feature rich soils and an extended growing season with sufficient rainfall or access to irrigation that are ideal for high value vegetable production. Moreover, in this temperate climate, vegetable harvest typically occurs from mid-Aug. to mid-Sept. allowing for sufficient time to grow a cover crop prior to winter.

Traditionally sown to provide ground coverage in periods of fallow, cover crops have the potential to improve soil quality as well as influence overall production in vegetable cropping systems [2]. Increased organic matter and improvements to soil health are documented benefits of long-term (> 5 yrs) cover crop residue addition [3,4]; reduced soil erosion and decreased nutrient leaching are considered immediate (≤ 1 yr) benefits of an established cover crop [5–7]. Autumn-planted cover crops are often implemented to scavenge mineral N [8–10], however, cover crop N release in the subsequent year, must be synchronized with main crop demand if N fertilizer requirements are to be effectively reduced [11]. Along with soil moisture and temperature, plant carbon to nitrogen (C/N) ratio is a determinant of residue decomposition that also depends on plant growth stage [12]. C/N is generally high for cereal grain crops when compared to legumes [13,14].

Autumn-planted cover crops, specifically those that become winter-killed, have not been extensively studied. Stivers-Young [15] evaluated several brassica and cereal cover crops planted post vegetable production in the Northern United States. They reported that all treatments decreased soil organic N in the autumn, however, brassica treatments that were left to winter-kill had the propensity for decomposition of biomass and N loss the following spring. A related cover crop study of soil aggregate breakdown, conducted in British Columbia, Canada, found that winter-killed barley (*Hordeum vulgare* L.) provided less improvement to aggregate stability when compared to treatments winter cereal rye or annual ryegrass (*Lolium multiflorum* Lam.) (cv. Westerwolds), but was still advantageous relative to bare soil [16]. As such, the potential benefits and or drawbacks of winter-killed cover crop in vegetable production need to be evaluated.

To date, few studies have explored the impact of different cover crop species on the insect pests and diseases of tomato. An Ontario, Canada study showed no impact on foliar or fruit diseases of tomato in response to cereal cover crops [17], while diseases caused by *Verticillium dahliae* or *Fusarium* spp. in California, USA [18] and incidence of anthracnose [*Colletotrichum coccodes* (Wallr.) S. Hughes] in Ohio, USA [19] were similarly unaffected by *Brassica* spp. cover crops. Thus, beyond crop yields and fruit quality there is a need to assess the role of cover crops on insect and disease pressure.

Growers tend to prioritize increased crop yields over reduced input costs [20]. Whether this benefit outweighs the costs of cover crops remains an unresolved question. There is considerable variation in the literature regarding the economic impacts of cover crops, as cover crops have contributed to increased profitability in some production systems but not in others [20,21]. The capacity to increase profitability through the use of cover crops may vary with factors such as location, type of cover crop, and type of main crop. This variation makes it difficult to draw conclusions regarding the profitability of cover crop systems in tomato production from existing literature and highlights the need for regional evaluations.

As growers must manage all aspects of crop production, there is a need to assess the impact of cover crops as a systems-based approach [22]. The objective of the study was to take an integrated approach to evaluate the impact of using winter cover crops for 3 yrs on processing tomato production in terms of 1) N contribution due to the cover crop, 2) insect and disease pressure under a grower-typical pest management program, and 3) fruit yield, quality, and profit margins with early and late cultivars. In addition to advancing knowledge of the medium-term impacts (3 yrs) of cover cropping, the systems-based approach will allow for informed recommendations to growers regarding cover crop selection.

## Materials and methods

### Experimental design

Cover crop trials were initiated in 2007 and 2008 at the University of Guelph Ridgetown Campus, Ridgetown, Ontario, Canada (42^o^46’N, 81^o^89’W) with cover crops grown each autumn as described by O’Reilly et al. [23,24] and Ouellette et al. [25]. The soil was a sandy loam Orthic Humic Gleysol (Typic Hapludalfs). The experiment was a split-split-plot factorial design with four replications in a processing pea (*Pisum sativum* L.) ‒ cover crop ‒ sweet corn (*Zea mays* L. var. *saccharata*) ‒ cover crop ‒ spring wheat (*Triticum aestivum* L.) ‒ cover crop ‒ tomato rotation [24]. Cover crop was the main factor; split-plot factors were preplant urea ammonium nitrate fertilizer (46:0:0) rate (0 or 140 kg N ha^-1^, designated starter N and full N treatment, respectively, see below as all plots received a small amount of N at transplanting) and tomato cultivar (early ‘TSH18’ or late ‘CC337’). Each main plot was 6 m by 16 m; split-plots were 6m (three twin rows) by 8m and the split-split plot 6 m wide by 4 m long. Tomatoes were transplanted at a density of 29,640 plants ha^-1^ with 15 kg N ha^-1^ applied within the transplant water. The starter N plots were included to evaluate whether the cover crop treatments were impacting immobilization/mineralization. Cover crop treatments were selected based on availability, relative cost, ease of planting and establishment, and regional importance. Treatments included a no cover crop control, oat, winter cereal rye (hereafter referred to as rye), oilseed radish (OSR), and mixture of OSR and rye (OSR+rye).

Following main crop harvests, crop residues were incorporated using a disk followed by a cultivator to prepare a fine seedbed of the entire trial area prior to sowing cover crop treatments in Aug. to early Sept. Oat, rye, OSR, and OSR + rye were sown using a drill at 80, 67, 16, and 9 + 34 kg ha^-1^, respectively. To ensure that the no cover crop control remained free of weeds, 810 g ae ha^-1^ glyphosate was applied to all control plots in the autumn three weeks after cover crop planting. The following spring within a week of cover crop biomass sampling, 1800 g ae ha^-1^ glyphosate was applied to the entire trial site to control weeds and to terminate rye cover crops. Approximately one week prior to tomato transplanting, the whole trial site was disked and cultivated. One day prior to transplanting, in the split-plots for the full N fertilized treatment, 140 kg N ha^-1^ of urea ammonium nitrate was broadcasted manually and the entire trial was cultivated. Tomatoes were transplanted on 26 May 2010 and 31 May 2011. Typical Ontario production practices for processing tomatoes, such as nutrient fertilization (except N) and other field management, were followed [26,27]. Precipitation was measured at an Environment Canada weather station less than 1 km from the experiment, where the 30-yr monthly total rainfall over the growing season was approximately 80 mm. Rainfall was 122 and 155 mm in May, 85 and 75 mm in June, 136 and 70 mm in July, 26 and 71 mm in Aug., and 21 and 53 mm in the first 15 d of Sept. in 2010 and 2011, respectively.

### Data collection

#### Cover crop biomass and C:N

With similar timings in the previous two years, cover crops were sown on 24 Aug. 2009 and 08 Sept. 2010 and aboveground biomass was collected on 02 Oct. 2009 and 04 Oct. 2010, and the following spring on 05 May 2010 and 05 May 2011 using two 0.25-m^2^ quadrats per plot. In the spring, residues remaining of winter-killed cover crops and aboveground biomass of rye cover crop were collected from two 0.25-m^2^ quadrats per plot. Fresh weights were recorded and biomass was dried at 60°C to constant weight, with data analyzed and reported on a dry weight basis. A representative cover crop subsample was ground to pass through a 1 mm screen using a Wiley mill (Thomas Scientific, Swedesboro, NJ, USA). Plant tissue N and C concentrations (%) were determined by combustion [28] using a LECO C/N determinator (Leco Corporation, St. Joseph, MI, USA). Plant tissue C and N content (kg N ha^-1^) were calculated as the product of concentration and plant biomass. Because each species in the cover crop mix was combusted individually, the C/N ratio was based on total C and N content in plant tissue for all cover crop treatments.

#### Soil baseline and N sampling

A day prior to tomato transplanting, a composite soil sample taken was from a minimum of twenty 3.5 cm diameter cores taken to 15 cm depth. Soil characteristics were determined according to Carter and Gregorich [29]. In 2010 soil pH was 6.6 (1:1 v/v method) with 3.8% organic matter (modified Walkley Black method), and a 62:22:16 sand:silt:clay texture (hydrometer method); 2011 soil was 6.6 pH, 2.5% OM, with a 72:25:3 texture. Soil P, K, Ca, and Mg content was 38, 188, 1719, and 150 mg kg^-1^ in 2010 and 30, 130, 1462, and 134 mg kg^-1^ in 2011, respectively; where P was determined according to the Olson bicarbonate extraction method and K, Ca, and Mg as per atomic absorption via ammonium acetate extraction. Cation exchange capacity, estimated based on ammonium acetate extraction and pH, was 11.5 MEQ 100 g^-1^ in 2010 and 9.91 MEQ 100 g^-1^ in 2011.

Within a day of tomato transplanting and harvest, soil mineral N content (NO_3_^‒^-N and NH_4_^+^-N) from each main- and split-plot, respectively, was determined for composite soil samples of at least five 3.5 cm diameter cores at 0 to 30, 30 to 60, and 60 to 90 cm depths. Soil sample mineral N quantification was conducted as described by Maynard et al. [30] using 2 M KCl extraction and a continuous segmented flow autoanalyzer (SEAL AutoAnalyzer III; Mequon, WI) using the cadmium reduction method and phenate method for NO_3_^—^N and NH_4_^+^-N, respectively. For all depths, soil mineral N was represented as the sum of NO_3_^—^N and NH_4_^+^-N expressed as kg N ha^-1^ based on soil bulk density measured in each plot using standard 89.3 cm^3^ rings in 2007 [24].

#### Tomato damage, yield, and quality

Foliar and fruit insect pests and diseases were monitored on 21 June, 07 July, 22 July, 26 July (tomato hornworm [*Manduca quinquemaculata* (Haworth)] only), and 13 Aug. in 2010 and on 04 July, 20 July, and 11 Aug. in 2011. Colorado potato beetle [*Leptinotarsa decemlineata* (Say)] (CPB) damage was evaluated by counting the number of plants with insect damage and estimating the percent defoliation per plot. Tomato hornworm damage was evaluated by counting the number of defoliated stems per plot. Damage per pest was distinguished by feeding pattern; CBP tends to feed all over the tomato plant, while hornworm damage typically involves the removal of leaf, petiole, and branch in a localized spot. Bacterial spot (*Xanthomonas vesicatoria*, *X*. *perforans*, *X*. *euvesicatoria*, *X*. *gardneri*), bacterial speck [*Pseudomonas syringae* pv. *tomato* (Okabe) Young, Dye, & Wilkie], bacterial canker [*Clavibacter michiganensis* subsp. *michiganensis* (Smith) Davis], early blight (*Alternaria solani* Sorauer (syns. *A*. *solani* (Ellis & G. Martin))), and Septoria leaf spot (*Septoria lycopersici* Speg.) symptoms on foliage were evaluated by counting the number of leaves with lesions on five plants per split-split-plot and estimating the leaf area affected by each disease.

When each split-split-plot reached > 80% maturity, 2 m was hand harvested from the center two rows of each split-split-plot. The early cultivar tomatoes were harvested on 25 Aug 2010 and 29–30 Aug 2011 and the late cultivar on 13–15 Sep. 2010 and 13–14 Sep. 2011. Fruits were individually separated into the following categories based on industry standards and a category weight taken: red fruit with < 5% yellowish exterior colour and free of defects that would render them culls; other marketable fruits with ≥ 50% of the surface area a blush colour and free of defects that would render them culls; unmarketable all remaining fruits. Random red fruit subsamples were allocated to determine fruit quality. Harvest area with fresh fruit and cull weights were used to calculate marketable and total yield (all fruit), respectively. To quantify N content, above ground biomass was collected from five representative plants, vegetative tissue separated from fruit, fruits were graded and each grade weighed. Vegetative tissue and 10% by weight subsample per fruit grade were each analyzed using the aforementioned N determination methods. A random representative sample of approximately 60 fruits was allocated to pest damage assessments.

At tomato harvest, the incidence of bacterial spot and bacterial speck on tomato fruit was evaluated by counting the number of affected fruit from a sample of 50 red (2011 only) and green fruit. The incidence of stink bug feeding damage was assessed from a random sample of 50 red fruit. Although *Euchistus servus* (Say), *E*. *variolarisus* (Palisot de Baeuvois), and *Acrosternum hilare* (Say) occur in Ontario, the specific stink bug species present in the trials were not identified as only feeding damage was evaluated and stink bugs were not observed during in-season scouting activities. Immediately following harvest, tomatoes were stored for three days at room temperature (~25°C) then evaluated for incidence of anthracnose. Fruits were sorted into classes 0 to 3, where 0 = no symptoms; 1 = one to two lesions, 2 = three to four lesions; and 3 = five or more lesions.

Fruit quality parameters were assessed on a random sample of at least 25 marketable fruit per plot. Fruits were washed, dried, and comminuted in a Waring CB6 commercial blender (Waring Commercial, Torrington, CT) on medium speed, under vacuum (88 kPa), for 40 s. The pulp was then passed through a 27 mesh screen to remove seed particles; colour was measured using an Agtron E-5M spectrophotometer calibrated at 48 [31,32], and pH was measured using a calibrated Orion pH meter (Thermo Fisher Scientific, Nepean, ON). Natural tomato soluble solids (NTSS) were measured on filtered (Fisherbrand P8 coarse porosity filter paper, Fisher Scientific, Pittsburgh, PA) pulp serum using a Palette PR101 temperature compensated digital refractometer (Atago USA, Inc., Bellevue, WA).

### Statistical analyses

Data were subject to ANOVA using PROC MIXED in SAS (SAS Institute, Cary, NC, ver. 9.1.) after satisfying the assumptions for normality and homogeneity of variance. Outliers were determined using the boxplot method and when outliers (i.e., those data points that were 1.5 times the interquartile range) were present, they were removed from the analysis only if results were significantly affected by their removal (i.e., a change in the significance of fixed effects) [33]. For any one parameter, only one to three (soil mineral N at 60–90 cm depth) data points were discarded (or missing) from analysis, which amounted to a grand total of nine data points for the entire dataset discussed in this paper. Data per year were analyzed separately due to multiple significant interactions of fixed effects with year as well as differences in sampling dates, and physical and environmental factors between the two years. Means were separated using the Tukey-Kramer adjustment test at the 0.05 probability level.

A split- split-plot analysis was used for all data except economic analyses, where cover crop type, N rate and cultivar, and their two-way and three-way interactions were fixed effects and replicate as random effect. A split-plot analysis was not required for the cover crop season because N rate and cultivar were not yet factors. For biomass and N content of cover crops, the control treatment was excluded in data analysis because there were no plants to sample.

### Economic analyses

The economic analysis compared profit margins over cover crop and fertilizer costs. These profit margins, which only take into account costs that vary among treatments, were calculated as total revenues less costs associated with cover crops and N application. Total revenues were calculated based on the marketable yield of tomato for each split-split-plot and the average prices offered by two local processors in 2010 and 2011. The costs associated with cover crops include seed costs, custom planting costs, herbicide costs for the treatments that included rye, and custom herbicide application costs. Seed costs for each type of cover crop were provided by seed retailers in southern Ontario. The cost of glyphosate used for the rye burndown was based on prices reported by AGRIS Co-operative Ltd (Chatham, Ontario). The costs of custom planting and herbicide application were determined based on the Ontario Ministry of Agriculture, Food, and Rural Affairs 2006 custom rate survey [34]. Nitrogen costs included the cost of fertilizer, which was based on prices charged by local fertilizer suppliers, and the cost of application, which was based on Ontario Ministry of Agriculture, Food, and Rural Affairs’ 2006 custom rate survey [34]. All other costs (e.g., processing tomato transplants, fertilizers, and pesticides applied to processing tomato) were assumed to be equal across treatments.

Tukey-Kramer was used to determine statistically significant differences in profit margins between treatments, while regression analysis was conducted to determine the impact of each cover crop on profit margins, relative to no cover crop. The regression equation was:

$$PM_{i}=a+b*Fert+c*Cultivr+d*Oat+e*OSR+f*OSRrye+g*Rye+\varepsilon_{i}$$

Where *PM*_*i*_ was profit margin over cover crop and N costs for plot *i*; *Fert* was a categorical variable equal to 1 for plots on which N was applied; *Cultivr* was a categorical variable equal to 1 if the early cultivar was planted; *Oat*, *OSR*, *OSRrye*, and *Rye* were categorical variables representing the use of each cover crop; *a*, *b*, *c*, *d*, *e*, *f*, and *g* were parameters to be estimated; and *ε*_*i*_ was the error term. The parameter estimate for each cover crop variable indicated the difference in profit margin from the treatment with no cover crop, all else equal. Both the pairwise comparisons and the regression analysis were initially conducted across all plots; subsequently, analysis was conducted separately for early and late cultivars as well as for treatments with N and treatments without, in order to examine for variation in the results across these factors.

## Results and discussion

### Cover crop, soil, and tomato N

In autumn 2009, cover crop biomass was greatest for OSR treatments (2.8 Mg ha^-1^). Oat and OSR + rye biomass were 1.7 and 1.8 Mg ha^-1^, respectively, yet rye grown alone was only 1.2 Mg ha^-1^. Nitrogen content for OSR, oat, OSR + rye, and rye was 70.8, 35.3, 42.3, and 27.7 kg N ha^-1^, respectively. Previously, O’Reilly et al. [24] found that N content was numerically highest for OSR, followed by OSR + rye, rye, than oat, when measured in autumn. In our present study, N concentration was not affected by cover crop and ranged from 2.0 to 2.5%. In terms of C/N ratios, oat was the greatest (21.1), while rye (17.0) was similar to OSR + rye (16.0), but higher than OSR (14.1).

Autumn 2010 cover crop planting was delayed by two weeks to best capture soil moisture conditions required for germination and establishment. Cover crop biomass was < 0.9 Mg ha^-1^ for all cover crops, with no significant difference, when sampled in early October. The cover crops likely continued to grow after sampling as the following spring, rye biomass (the only treatment cover crop that over-winters) was 2.7 to 3.1 Mg ha^-1^. From the same long-term cover crop experiment, when the same four cover crop were sampled in late Oct. to early Nov., the 3-yr average cover crop biomass was 3.1 to 3.6 Mg ha^-1^; N concentration of 1.6 to 2.6%; and C/N ratio of 16.2 to 25.3 [25]. Likewise, comparable cover crop biomass and N content values have been observed for studies conducted in a temperate climate, as in the current study [23,35–37].

By spring, cover crop biomass for rye-containing treatments (i.e. rye and OSR+rye treatments, where OSR residue was included) was relatively high (2.7 Mg ha^-1^), yet oat (1.8 Mg ha^-1^) and OSR (2.6 Mg ha^-1^) were similar to autumn sampling, indicating very little decomposition. Oilseed radish N concentration was 1.0% in the spring (compared to ≥ 2.5% in the autumn), while the remaining cover crops ranged from 1.7 to 1.8%, suggesting that OSR may have undergone N leaching from residues [38] and perhaps more N release from OSR biomass than other cover crops. This was consistent with Miller et al. [38], who reported that over 10% biomass N (NO_3_^—^N or NH_4_^+^-N) was leached from OSR under a simulated rainfall study. Additionally, Schomberg et al. [39] found that rate of spring mineralization was up to 50% slower for rye when compared to OSR that had been sown the previous autumn. Cover crop C/N ranged from 19.9 to 23.8 and thus all were considered favorable for mineralization. When compared to autumn values, in the following spring C/N was the same for oat but OSR, OSR + rye, and rye treatments each increased by ≥ 5, indicating decomposition of OSR and the transition of rye to reproductive stage of development. The range of C/N values of cover crop biomass was in agreement with those observed elsewhere [36,40]. The observed quantity and quality (C/N) of cover crop biomass was supported by Ouellette et al. [25] who reported that OSR and OSR + rye cover crops have the potential to increase labile C pools in ≤ 4 yrs.

Prior to tomato planting, soil mineral N at all depths was affected by cover crop type, with the exception of 2011 shallow samples (0 to 30 cm) (Table 1). In general, the trend OSR ≥ oat ≥ no cover crop ≥ OSR + rye = rye was observed for soil preplant mineral N at all depths (Table 1). Although OSR had 14.1 and 23.6 kg N ha^-1^ higher soil mineral N (0–60 cm depth) than the no cover crop control in spring 2010 and 2011, respectively, it is unlikely that processing tomato growers should modify their N applications based on preplant soil mineral N content. Likewise, others [24,35,41] reported that cover crops OSR, oat, and rye did not enhance N availability to a successional corn crop.

**Table 1:** Soil mineral N (nitrate-N and ammonium-N) at pre-tomato transplanting from three sampling depths in 2010 and 2011^a^.

	2010			2011		
Cover crop treatment ^b^	0–30 cm	30–60 cm	60–90 cm	0–30 cm	30–60 cm	60–90 cm
	kg N ha^-1^					
No cover crop	14.5 b	9.44 b	6.58 b	11.9	13.2 b	8.46 c
Oat	15.1 b	10.2 b	4.23 bc	14.4	26.2 a	15.5 ab
OSR	18.9 a	19.1 a	11.1 a	17.3	31.4 a	19.0 a
OSR + rye	17.0 ab	5.96 c	2.40 dc	14.2	16.2 b	6.42 c
Rye	14.2 b	3.62 c	1.04 d	13.8	9.40 b	10.0 bc
SE	1.742	0.899	0.733	2.049	3.136	1.589
*P* value	**0.0026**	**<0.0001**	**<0.0001**	0.4972	**0.0005**	**0.0013**

^a^ Different letters in each column indicates a statistical difference, P ≤ 0.05, Tukey-Kramer adjustment.

^b^ Cover crop treatments were planted the autumn preceding tomato production. OSR, oilseed radish; rye, winter cereal rye.

In 2010 at tomato harvest, a significant N rate by cultivar interaction was identified in shallow depth (0 to 30 cm) that could be attributed to 14.1 kg N ha^-1^ more with the full N rate compared to starter N in the early cultivar; however, no corresponding difference was observed in late cultivar (S1 Fig). There was also a cover crop by cultivar interaction in 2010 in the medium soil depth (30 to 60 cm), which was due to approximately 10 kg N ha^-1^ higher soil mineral N with OSR in the early cultivar compared to all other cover crop treatments (S1 Fig). But with the late cultivar, oat was approximately 5 kg N ha^-1^ higher than the other cover crops. It is not known why these interactions occurred but may be due to differences in cover crop root depths [42] and timing of mineralization.

Aside from deep (60 to 90 cm) depth soil samples in 2010, soil mineral N was affected by cover crop treatment at tomato harvest (Table 2). All treatments except rye increased soil mineral N at 0 to 30 cm in 2011, when compared to the no cover crop control (Table 2). For 2010 shallow and 2011 medium soil depth samples, only OSR increased soil mineral N, while none of the cover crops evaluated were different from the control at 60 to 90 cm in 2011 (Table 2). Similarly, O’Reilly et al. [24] assessed soil mineral N during the sweet corn growing season and found that although few significant differences were present, OSR tended to increase N whereas OSR + rye, rye, and oat cover cops reduced N. Soil mineral N in the 0 to 30 cm depth for full N was 7.6 kg ha^-1^ more than starter N in 2010 (Table 2).

**Table 2:** Soil mineral N (nitrate-N and ammonium-N) at tomato harvest from three soil depths in 2010 and 2011^a^.

	2010			2011		
	0–30 cm	30–60 cm	60–90 cm	0–30 cm	30–60 cm	60–90 cm
	kg N ha^-1^					
Cover crop [CC]^b^						
No cover crop	18.9 b	8.54	5.45	21.0 c	7.17 c	6.96 ab
Oat	16.8 b	11.3	5.65	29.4 a	13.7 ab	6.68 b
OSR	26.4 a	12.6	6.83	28.2 ab	17.3 a	12.0 a
OSR + rye	21.0 ab	9.24	5.23	29.3 a	11.8 abc	9.27 ab
Rye	19.5 b	7.92	5.63	24.2 bc	11.4 bc	6.19 b
SE	2.54	1.490	1.047	3.80	1.514	1.589
N rate [N]						
Starter N	16.7 k	9.30	5.98	25.4	11.9	8.63
Full N	24.3 j	10.6	5.53	27.3	12.7	7.82
SE	1.91	0.942	0.833	3.369	0.997	1.208
Cultivar [C]						
Early	28.3 z	15.9 z	9.52 z	23.5 y	12.7	9.75 z
Late	12.7 y	3.98 y	1.99 y	29.3 z	11.9	6.71 y
SE	1.91	0.942	0.833	3.369	0.997	0.208
Effect	*P* value					
CC	**0.0377**	0.1457	0.6830	**0.0388**	**0.0003**	**0.0142**
N	**0.0003**	0.3436	0.5429	0.3360	0.5815	0.4987
C	**<0.0001**	**<0.0001**	**<0.0001**	**0.0056**	0.5328	**0.0134**
N*C	**0.0015** ^c^	0.0723	0.1032	0.7164	0.7822	0.8591
CC*N	0.1697	0.4786	0.9150	0.2904	0.7862	0.1119
CC*C	0.1157	**0.0106** ^d^	0.3125	0.9316	0.8059	0.4788
CC*N*C	0.1100	0.8126	0.8684	0.8380	0.9360	0.9043

^a^ Different letters in each column indicates a statistical difference, P ≤ 0.05, Tukey-Kramer adjustment.

^b^ Cover crop treatments were planted the autumn preceding tomato production. OSR, oilseed radish; rye, winter cereal rye.

^c^ Interaction due to higher soil mineral N with the full N rate compared to starter N in the early cultivar only (S1 Fig).

^d^ Interaction due to higher soil mineral N with OSR than all other treatments with the early cultivar but with the late cultivar, soil mineral N was higher with oat than the other cover crops (S1 Fig).

Nitrogen concentration of tomato plants was not affected by cover crop treatment, ranging from 2.4 to 2.9% N in fruits and 1.2 to 1.7% N in shoots (Table 3). Cover crop by cultivar interaction was detected for shoot N content in 2010 that can be explained by OSR late cultivar shoots containing least 9 kg N ha^-1^ more than the other treatments (S2 Fig). Despite tomato fruit and shoot N content for OSR being greater than oat and rye, no individual treatment was different than the no cover crop control in 2010 (Table 3). This subtle difference was not unexpected as no significant impact on fruit or shoot biomass were present for any cover crop treatment in 2010 or 2011 (data not shown). In 2011, fruits and shoots for OSR were 20 and 10 kg N ha^-1^ greater than the control, respectively; fruits with oat or rye cover crops were at least 10 kg N ha^-1^ more (Table 3). Nitrogen rate directly impacted tomato N, where N content and N concentration of fruit and shoot tissue were greater for the full N rate (Table 3). Lenzi et al. [40] found similar tomato fruit N content values, but in contrast to our observations, they observed no significant difference among non-legume cover crops and the no cover crop control treatments.

**Table 3:** Nitrogen concentration (%) and content (kg ha^-1^) in tomato fruit and shoot tissue in 2010 and 2011^a^.

	Fruit				Shoot			
Treatment	2010		2011		2010		2011	
Cover crop [CC]	%	kg ha^-1^	%	kg ha^-1^	%	kg ha^-1^	%	kg ha^-1^
No cover crop ^b^	2.74	106 ab	2.50	117 c	1.63	29.1 ab	1.26	19.6 b
Oat	2.62	95.6 b	2.61	133 ab	1.46	23.7 b	1.33	27.0 ab
OSR	2.85	114 a	2.78	137 a	1.67	32.7 a	1.45	29.9 a
OSR + rye	2.57	105 ab	2.58	117 c	1.60	26.1 ab	1.37	28.5 ab
Rye	2.74	96.6 b	2.44	129 b	1.48	23.7 b	1.23	22.0 ab
SE	0.093	9.58	0.114	5.6	0.065	2.51	0.058	2.65
N rate [N]								
Starter N	2.58 j	94.4 j	2.46 j	109 j	1.47 j	23.8 j	1.24 j	20.5 j
Full N	2.81 k	106 k	2.70 k	145 k	1.64 k	29.3 k	1.42 k	30.3 k
SE	0.066	8.90	0.072	3.8	0.047	2.15	0.037	1.99
Cultivar [C]								
Early	3.36 y	99.9	2.56	117 z	1.82 y	25.3 z	1.36	24.7
Late	2.03 z	106	2.60	137 y	1.29 z	27.8 y	1.31	26.1
SE	0.066	8.90	0.072	3.8	0.047	2.15	0.037	1.99
Effect	*P* values							
CC	0.1607	**0.0384**	0.2884	**0.0169**	0.0924	**0.0025**	0.0706	**0.0083**
N	**0.0137**	**0.0016**	**0.0240**	**<0.0001**	**0.0101**	**0.0046**	**0.0007**	**<0.0001**
C	**<0.0001**	0.2509	0.6709	**<0.0001**	**<0.0001**	0.1913	0.3392	0.4460
N*C	0.4796	0.1911	0.5847	0.2706	0.2002	0.9912	0.7231	0.5368
CC*N	0.7534	0.6500	0.6164	0.7106	0.8711	0.3447	0.2612	0.1162
CC*C	0.5741	0.2098	0.7986	0.0569	0.4053	**0.0229** ^c^	0.9552	0.9529
CC*N*C	0.6020	0.8105	0.8887	0.0825	0.7918	0.1982	0.3920	0.4128

^a^ Different letters in each column indicates a statistical difference, P ≤ 0.05, Tukey-Kramer adjustment.

^b^ Cover crop treatments were planted the autumn preceding tomato production. OSR, oilseed radish; rye, winter cereal rye.

^c^ Interaction due to higher values with OSR in the late cultivar than all other treatments (S2 Fig).

### Insect and disease damage

During the tomato growing season, the severity of foliar diseases (bacterial spot and speck, bacterial canker, early blight, and septoria leaf spot) and incidence of foliar insect damage caused by CPB and tomato hornworm, were well below established economic thresholds (data not shown). There were no differences among cover crop, N rate, or cultivar treatments for any of the aforementioned pests (S1 Table). The causal agents of bacterial speck, bacterial canker, early blight, and septoria leaf spot overwinter on crop residue or alternate hosts under Ontario growing conditions [43–46]. Similarly, CPB overwinters in soil, travels short distances to host crops, and affects only members of the *Solanaceae* plant family [47]. Thus, considering the pest life cycles, overall disease and CPB pressure in the area during the two growing seasons was considered low. Additionally, tomato crops in this study were treated aggressively with preventative fungicides and insecticides, which further limited the development of fungal pathogens and insect pests. Therefore, based on the data set it is not possible to conclude whether cover crops without a pesticide management program can influence the incidence or severity of common foliar tomato pests in Ontario.

No significant differences were detected for cover crop or N rate for bacterial spot and speck on tomato fruit in either year (S1 Table). However, there was a cultivar effect; 5.7 and 43.5% of early and late green fruits in 2010 had lesions, respectively. This difference could be an indication that conditions for infection were more favorable later in the season or a potential cultivar-specific sensitivity to bacterial spot and speck. In 2011, incidence of bacterial spot and speck was low, affecting 4.1 to 7.6% of red fruit and levels in green fruit were similar, with no differences in cover crop, N rate, and cultivar. Likewise, fruit damage due to stink bug was low (≤ 7%) and showed no differences for any treatment factors. With the exception of 2011 cultivar, no significant differences were present for anthracnose incidence on fruit for cover crop, N rate, and cultivar in both years. In 2011, 7.9 and 27.2% of early and late fruits were affected with anthracnose. This may be an indication that in 2011, conditions during peak bloom and initial fruit set for the late cultivar were more favorable for infection by anthracnose than during the same growth stage for the early cultivar; regardless there was no cover crop treatment effect. Similar to our study, Summers et al. [48] found inconsistent effects of cover crops on tomato disease levels (i.e., 0–44% of trials), and thus could not make general cover crop recommendations. Overall, a lack of cover crop effect or significant interaction with N rate or cultivar suggests that the cover crops did not exaggerate or minimize insect or disease damage on fruit when used with a typical pesticide program.

### Processing tomato yield

In 2010 and 2011, processing tomato marketable yields for the full N rate (i.e., across cover crops and cultivar) were 106 and 120 Mg ha^-1^, respectively. Yields were representative of commercial growers (K. Evans, Conagra Foods Canada Inc. pers. comm.) but higher than reported provincial average yield of 78.7 Mg ha^-1^ in 2008 to 2012 [49].

A cover crop by N rate interaction was present for 2011 marketable yields that was due to an increase in yields with full N for all other cover crop treatments, but no difference between N rate for OSR and OSR + rye (S3 Fig). This trend was also observed with total and red yields in 2011. The cover crop by N rate interaction suggests differences among cover crop treatments in soil N dynamics. Relative to the no cover crop control, no individual cover crop treatment increased tomato yield in 2010 (Table 4). In 2011, total and marketable tomato yields were greatest for OSR (Table 4). Yield of red fruit was greatest for OSR and rye cover crops; however, OSR + rye did not differ from the control (Table 4). In all cases, yields with a cover crop were as good as or better than without a cover crop. Like the results of this study, others have observed similar or higher tomato yields with cover crops than without [40,50,51]. For instance, Sainju et al. [52] reported that tomato yield following rye cover crops did not differ from an unfertilized no cover crop control. An inconsistent impact of cover crops on tomato yields was observed by Summers et al. [48], where only 4 of the 16 sites displayed a significant cover crop effect. The mechanism to explain cover crop-derived increases in tomato yield is not known but is presumably soil mediated. Some attribute the cover crop effect on tomato yield to increased soil microbial C and N associated with incorporation of cover crop residues [50,51].

**Table 4:** Impact of cover crop, N rate and cultivar on processing tomato yields in 2010 and 2011^a^^b^.

	2010			2011		
Treatment	Total	Marketable	Red	Total	Marketable	Red
Cover crop [CC] ^c^	Mg ha^-1^					
o cover crop	106 ab	100 ab	83.2	107 b	104 b	88.5 b
Oat	102 ab	95.7 ab	79.9	118 ab	114 ab	98.8 ab
OSR	114 a	103 ab	83.8	124 a	118 a	101 a
OSR + rye	111 a	104 a	86.4	110 ab	106 ab	92.8 ab
Rye	95.9 b	91.2 b	79.2	114ab	113 ab	102a
SE	6.76	6.60	5.37	3.6	3.2	3.07
N rate [N]						
Starter N	103	96.8	81.2	104 k	102 k	88.2 k
Full N	109	101	83.8	125 j	120 j	105 j
SE	6.1	6.02	4.84	2.4	2.2	1.94
Cultivar [C]						
Early	88.1 z	80.6 z	56.7 z	107 z	103 z	80.1 z
Late	124 y	117 y	108 y	122 y	119 y	113 y
SE	6.14	6.00	4.84	2.4	2.2	1.94
Effect	*P* values					
CC	**0.0057**	0.0601	0.4238	**0.0093**	**0.0131**	**0.0092**
N	0.0861	0.1970	0.3368	**<0.0001**	**<0.0001**	**<0.0001**
C	**<0.0001**	**<0.0001**	**<0.0001**	**<0.0001**	**<0.0001**	**<0.0001**
N*C	0.7580	0.5762	0.2929	0.5737	0.4662	0.4150
CC*N	0.7476	0.6384	0.6189	0.0559	**0.0322** ^d^	0.0638
CC*C	0.1489	0.1835	0.1490	0.8471	0.8755	0.8662
CC*N*C	0.3537	0.2706	0.5314	0.7805	0.5623	0.8079

^a^ Different letters in each column indicates a statistical difference, P ≤ 0.05, Tukey-Kramer adjustment.

^b^ Marketable fruit includes red fruit category. Total fruit was marketable and unmarketable fruit.

^c^ Cover crop treatments were planted the autumn preceding tomato production. OSR, oilseed radish; rye, winter cereal rye.

^d^ Interaction due to higher values with full N for all other cover crop treatments, but no difference between N rate for OSR and OSR + rye (S3 Fig).

Applying full N increased total, marketable, and red tomato yields in 2011, with numerical but not statistical increases in 2010 (Table 4). The lack of N effect in 2010 may be due to low precipitation in July and August that limited full yield potential. Relatively high yields with the starter N treatment, in both years, was reflective of fertile, productive soil with relatively high organic matter content for the sandy loam texture. The late cultivar consistently yielded greater than the early cultivar, notably in 2010 where red fruit yields were nearly doubled (Table 4). A literature review of cover crops grown in temperate climates found that the presence of cover crops tends to increase or have no effect on subsequent yields, except where water is limiting where cover crops tend to reduce yields [22]. It is difficult to make direct comparisons to the existing literature as few employ autumn-sown cover crops whose residues are fully incorporated prior to tomato production.

### Fruit quality

There was no effect of cover crop type, N rate, nor any two- or three- way interactions for fruit quality parameters of Agtron colour or pH (S2 Table). There was a significant cultivar effect for Agtron colour (17.8 and 19.2 for early and late cultivars, respectively) and pH (4.27 and 4.40 for early and late cultivars), which would be expected due to genotype differences. All values were within commercially acceptable values for processing tomatoes [32,53]. Similarly, Seliga and Shattuck [54] observed tomato colour was not impacted by previous crop, N rate (in 1 of 2 yrs) or their interaction. Likewise, others have seen no effect of cover crop treatment on tomato colour [36] or pH [36,40,55]. The lack of cover crop effect and cover crop interactions with N rate or cultivar interaction suggests limited impact of adopting cover crops into tomato production practices based on Agtron colour and pH fruit quality.

The measurements of NTSS were all within the historic range for Ontario tomatoes grown for processing [56]. The main effect of cover crop treatment was not significant for NTSS (S2 Table), which was similar to other cover crop studies [40]. Similarly, previous studies observed that crop rotation, which included red clover (*Trifolium pretense* L.) cover crop, had no influence on NTSS in processing tomato [54]. Thus, our results were consistent with those reported in the literature.

Differences in NTSS were detected in only the cultivar main effect and the cultivar-by-cover-crop interaction (S2 Table). The cultivar-by-cover crop interaction was due to a difference in NTSS only between the early and late cultivar in the OSR + rye cover crop of 4.03 and 4.34, respectively. In all other cover crop treatments there were no differences detected between the early and the late cultivar for NTSS, nor between any of the other cultivar-by-cover crop treatment combinations. The OSR + rye treatment tended to emphasize some difference between cultivar and increase divergence in NTSS levels over the other cover crop treatments. Further studies would be needed to determine individual cultivar response under this production management practice if maximizing NTSS is a priority.

### Economics

The results of the pairwise comparisons (Table 5) indicated that across all plots the OSR and oat treatments had significantly greater profit margins than that of the no cover crop control, which suggests that the use of these cover crops can increase profit margins. Among the cover crops tested, the OSR + rye treatment had the lowest profit margin, which was significantly lower than the OSR treatment. Profit margins were greater for the late cultivar than for the early cultivar, due to higher yields for the late cultivar, and in both cases, only the OSR treatment had significantly greater profit margins relative to the no cover crop control. For treatments with full N, OSR + rye had the lowest profit margin but for treatments with starter N all cover crop treatments except for rye had significantly higher profit margins than the no cover crop control. For each of the sets of results in Table 5 (i.e., all plots; early cultivar; late cultivar; full N; starter N), the differences in profit margins are attributable to difference in cover crop treatment, as all other factors are either accounted for in the analysis or held constant across treatments. Overall, these results indicated that economic benefits may exist with the use of cover crops before tomatoes, particularly with OSR and oat. Similar to our results, neutral or favourable profit margins in sweet corn production were observed with cover crops [24].

**Table 5:** Processing tomato profit margins over N rate and cover crop costs, broken down by treatment ($ ha^-1^) in 2010 and 2011^a^.

Cover crop treatment^b^	Combined	Early cultivar	Late cultivar	Full N	Starter N
No cover crop	10,450 c	9,713 b	11,190 b	11,860 a	9,051 b
Oat	11,410 ab	10,630 ab	12,190 ab	12,320 a	10,510 a
OSR	11,770 a	10,980 a	12,570 a	12,190 a	11,350 a
OSR + rye	10,550 bc	9,378 b	11,720 ab	10,600 b	10,500 a
Rye	11,210 abc	10,340 ab	12,080 ab	12,280 a	10,140 ab

^a^ Different letters in each column indicates a statistical difference, P ≤ 0.05, Tukey-Kramer adjustment.

^b^ Cover crop treatments were planted the autumn preceding tomato production. OSR, oilseed radish; rye, winter cereal rye.

The results of the regression analysis (Table 6) for all cultivars and N rates indicated that profit margins were $1,320 greater for the OSR treatment and $960 greater for the oat treatment than for the no cover crop control treatment. While there are positive parameter estimates for the rye and the OSR + rye treatments, these differences from the control treatment were not statistically significant. The use of full N fertilizer was found to increase profit margins by over $1,500, while the use of the early cultivar was found to reduce margins by over $1,700, relative to the late cultivar. For both the early cultivar and late cultivar, OSR increased profit margins by at least $1,200 relative to the no cover crop control, though these differences were only significant at the 10% level. For the treatments with full N, the OSR +rye treatment was found to reduce profit margins by over $1,200, relative to the control treatment. For treatments with starter N, the OSR treatment increased profit margins by just over $2,300 relative to the control, while both the oat and OSR + rye treatments increased profit margins by over $1,400. Overall, these results, which are consistent with those of the pairwise comparisons, suggest that the net economic benefit of cover crops prior to planting a tomato crop was greatest and most statistically significant for OSR, while the use of an oat cover crop also provided a net economic benefit. It is evident from these results that variation exists across types of cover crops in relative profitability, which was similar to those conclusions drawn in literature reviews [20–22]. In contrast to our study and O’Reilly et al. [23,24] which report favourable profit margins of fresh market tomato production with cover crops, Ding et al. [57] found that hairy vetch cover crop as a mulch was not economically viable. In their review, Blanco-Canqui et al. [22] identify the need for economic assessments of cover crops as these studies are limited.

**Table 6:** Regression analysis of processing tomato profit margins in 2010 and 2011 ^a^.

Variable	All Plots		Early cultivar		Late cultivar		Full N		Starter N	
	Coefficient	SE	Coefficient	SE	Coefficient	SE	Coefficient	SE	Coefficient	SE
Constant	10,550**	386.22	9,046**	510.09	10,320**	525.77	12,830**	453.81	9819**	508.44
Nitrogen	1,540**	291.96	1,333**	416.48	1,750**	429.29				
Early	-1,744**	291.96					-1,952**	370.53	-1,535**	415.14
Oat	960.20*	461.63	920.20	658.52	1,000	678.77	467.20	585.87	1,453*	656.39
OSR^b^	1,321**	461.63	1,266†	658.52	1,376†	678.77	339.20	585.87	2,303**	656.39
OSR + rye	96.78	461.63	-334.90	658.52	528.50	678.77	-1,251*	585.87	1,445*	656.39
Rye	754.40	461.63	624.60	658.52	884.20	678.77	422.30	585.87	1,086	656.39

†, *, ** indicate statistical significance at P ≤ 0.10, 0.05, 0.01, respectively.

^a^ Dependent variable was profit margins over cover crop and N costs.

^b^ Cover crop treatments were planted the autumn preceding tomato production. OSR, oilseed radish; rye, winter cereal rye.

## Conclusions

As a general principle of soil management, leaving plant residues in the soil has a positive influence on overall crop production [21,36,58]. None of the cover crops tested had a negative influence on tomato fruit yield, quality or presence of /damage from common pests under a typical pest management program. Despite OSR cover crops increasing soil mineral N prior to tomato transplanting and at harvest, the change was modest and did not consistently result in increased processing tomato yield compared to starter N with the full N rate. As such, growers would not likely need to modify N rates based on cover crop type. An economic analysis, which included the cost to hire a custom applicator to plant and to control winter, cereal rye in the spring, showed favorable profit margins for OSR. None of the cover crops decreased yield or profit margins compared to the no cover crop control, with OSR and oat showing the greatest profit margins. Therefore, all of the cover crops evaluated can be considered potential autumn-sown options prior to processing tomato production based on a systems-based approach. These results were observed on a field with good tilth soil (sandy loam, >2.5% organic matter), perhaps greater differences would be observed on degraded soils.

## Supporting information

S1 Fig Interactions on cover crop, fertilizer N rate, and cultivar (early, late) on soil mineral nitrogen in 0–30 cm (top) and 30–60 cm (bottom) at tomato harvest in 2010. OSR, oilseed radish; rye, winter cereal rye; starter N, no preplant fertilizer N applied; Full N rate, urea ammonium nitrate (140 kg N ha^-1^) preplant broadcast incorporated.
(TIF)

S2 Fig Interaction of cover crop, fertilizer N rate and processing tomato cultivar (Early, Late) on tomato shoot biomass at harvest in 2010. OSR, oilseed radish; rye, winter cereal rye; 0N, Starter N only treatment; FullN, urea ammonium nitrate (140 kg N ha^-1^) preplant broadcast incorporated.
(TIF)

S3 Fig Interaction of cover crop treatment and fertilizer N rate on marketable processing tomato yield in 2011. OSR, oilseed radish; rye, winter cereal rye; StarterN only, no preplant fertilizer N applied; Full N rate, urea ammonium nitrate (140 kg N ha^-1^) preplant broadcast incorporated.
(TIF)

S1 Table Impact of cover crop, fertilizer N rate, and cultivar on tomato foliar and fruit insect and disease damage in 2010 and 2011. OSR, oilseed radish; rye, winter cereal rye.
(PDF)

S2 Table Impact of cover crop, N rate and cultivar on processing tomato quality in 2010 and 2011. NTSS, natural tomato soluble solids; OSR, oilseed radish; rye, winter cereal rye.
(PDF)

## Abbreviations

CPB: Colorado potato beetle
NTSS: natural tomato soluble solids
OSR: oilseed radish
