# Supplementary figures and images for: Winter cover crops on processing tomato yield, quality, pest pressure, nitrogen availability, and profit margins
Source: PLoS One. 2017 Jul 6;12(7):e0180500. doi: 10.1371/journal.pone.0180500 (PMC5500340; doi:10.1371/journal.pone.0180500)

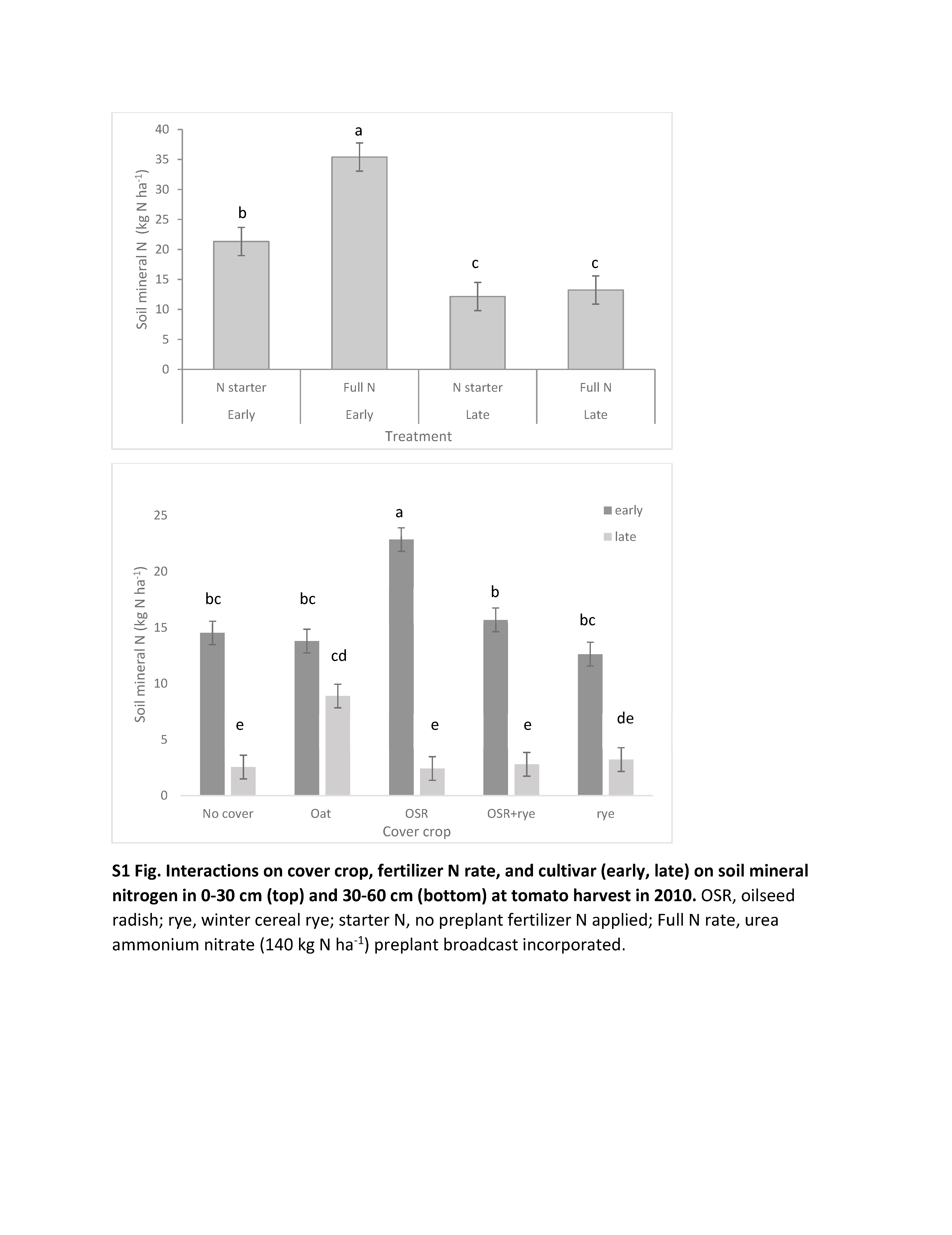

Supplement: S1 Fig — OSR, oilseed radish; rye, winter cereal rye; starter N, no preplant fertilizer N applied; Full N rate, urea ammonium nitrate (140 kg N ha-1) preplant broadcast incorporated. (TIF) [file pone.0180500.s001.tif]

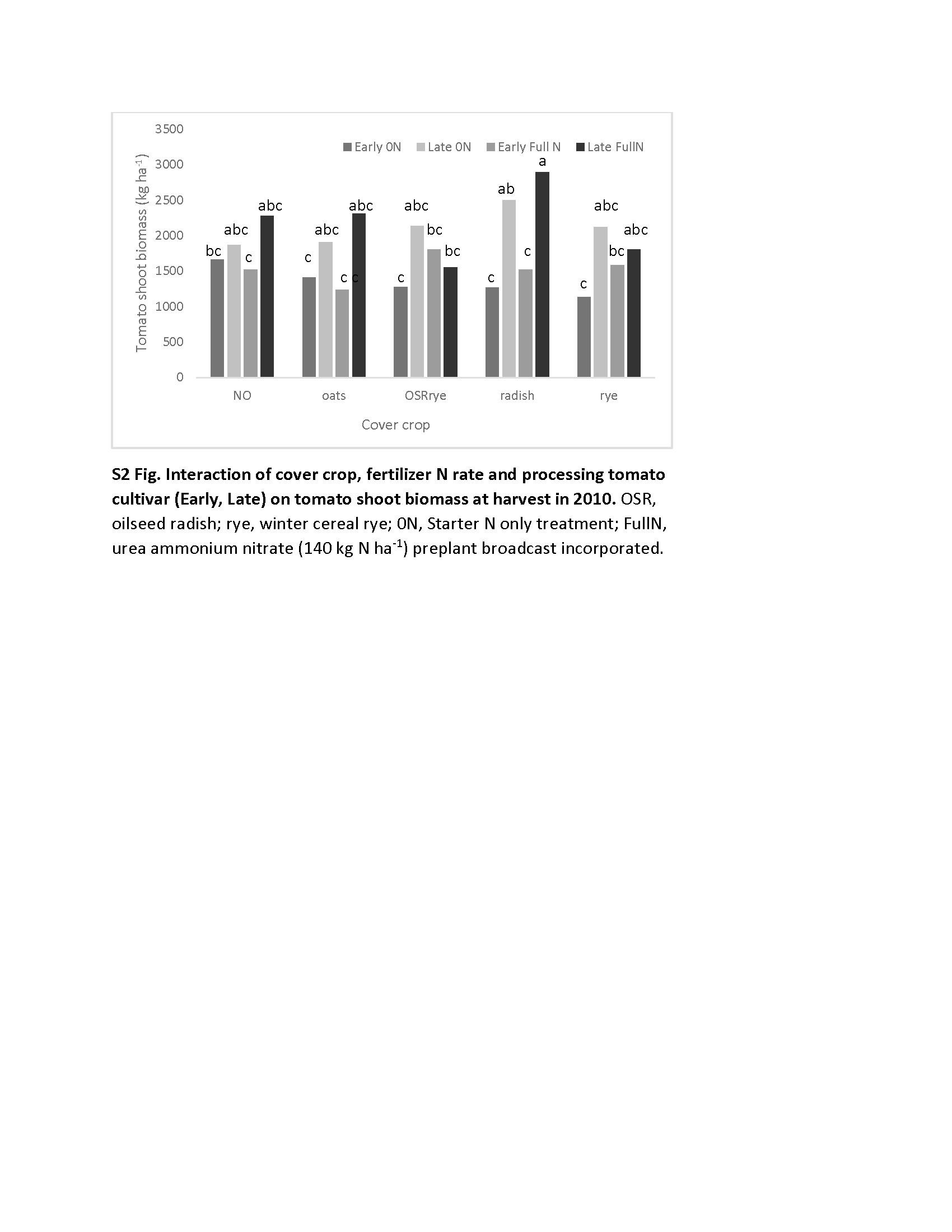

Supplement: S2 Fig — OSR, oilseed radish; rye, winter cereal rye; 0N, Starter N only treatment; FullN, urea ammonium nitrate (140 kg N ha-1) preplant broadcast incorporated. (TIF) [file pone.0180500.s002.tif]

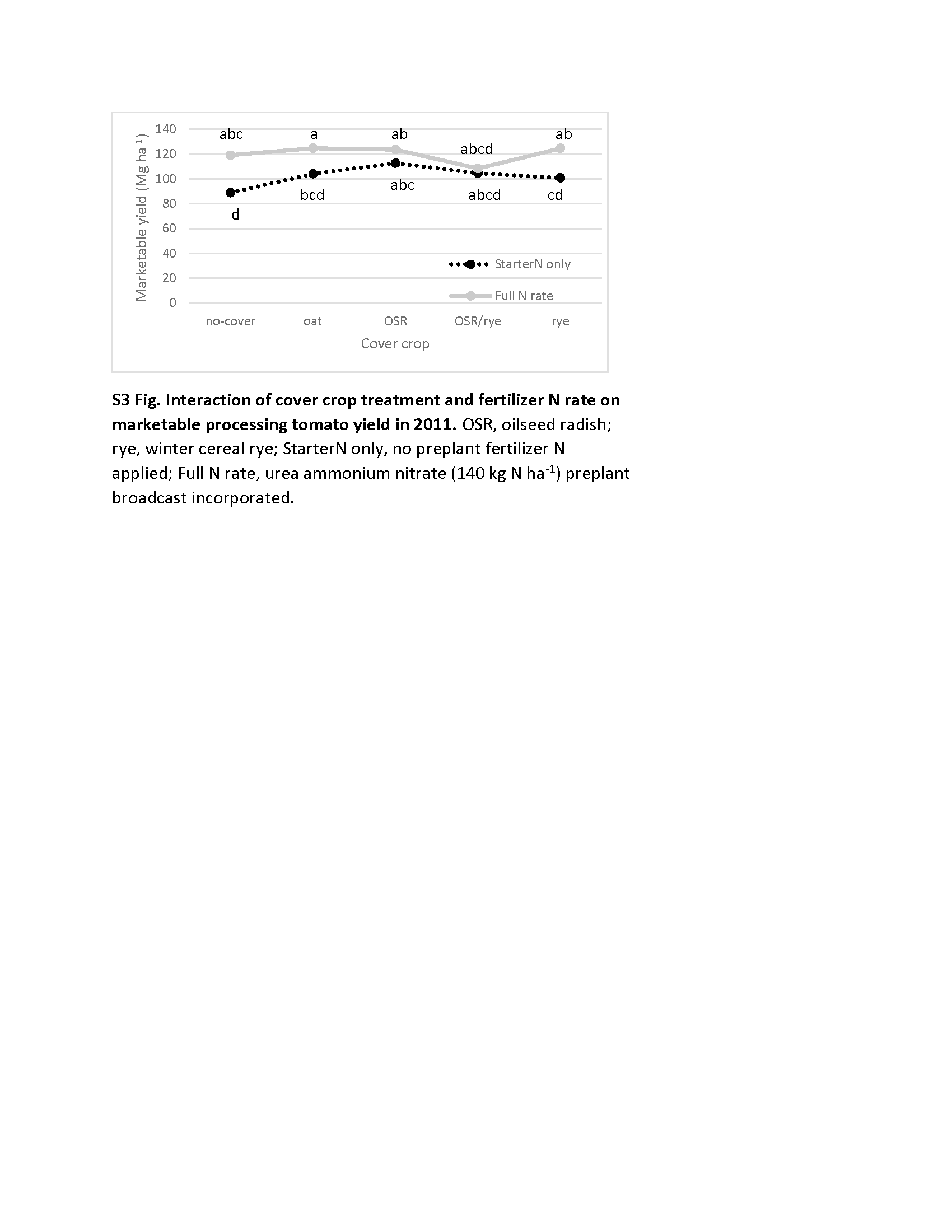

Supplement: S3 Fig — OSR, oilseed radish; rye, winter cereal rye; StarterN only, no preplant fertilizer N applied; Full N rate, urea ammonium nitrate (140 kg N ha-1) preplant broadcast incorporated. (TIF) [file pone.0180500.s003.tif]
