# Supplementary material for: Winter cover crops on processing tomato yield, quality, pest pressure, nitrogen availability, and profit margins
Source: PLoS One. 2017 Jul 6;12(7):e0180500. doi: 10.1371/journal.pone.0180500 (PMC5500340; doi:10.1371/journal.pone.0180500)
Supplement: S1 Table — OSR, oilseed radish; rye, winter cereal rye. (PDF) [file pone.0180500.s004.pdf]

S1 Table. Impact of cover crop, fertilizer N rate, and cultivar on tomato foliar and fruit insect and disease damage in 2010 and 2011. OSR, oilseed radish; rye, winter cereal rye.

S1a Table. Impact of cover crop, fertilizer N rate, and cultivar on bacterial disease incidence on tomato foliage in 2010 and 2011.

	# Infected leaves on 5 plants[a]					
	Bacterial Spot / Speck				Bacterial Canker	
	2010	2011			2010	
**Cover crop** [b]	June 21	Aug 8[c]	July 4[c]	July 20[c]	July 22[c]	Aug 8[c]
OSR	0.0 ns[d]	0.6 ns	0.1 ns	0.0 ns	0.2 ns	1.8 ns
OSR + rye	0.0	2.6	0.4	0.1	0.2	1.0
No cover crop	0.0	0.4	0.2	0.0	0.0	0.6
Oat	0.0	2.8	0.1	0.1	0.0	0.9
Rye	0.0	1.4	0.3	0.0	0.0	0.6
**N fertilizer to tomatoes**						
Starter N only	0.0 ns	1.8 ns	0.2 ns	0.0 ns	0.1 ns	1.4 ns
Full N	0.0	1.3	0.3	0.1	0.1	0.8
**Cultivar**						
Early	0.0 ns	2.0 ns	0.4 b	0.1 ns	0.0 ns	1.2 ns
Late	0.0	1.1	0.1 a	0.0	0.2	0.9

[a] The number of infected leaves or fruit on five plants was recorded.
[b] Cover crop treatments were planted the autumn preceding tomato production. OSR, oilseed radish; rye, winter cereal rye.
[c] Data was not normal and could not be normalized using square root or log transformation.
[d] Different letters in each column indicates a statistical difference, P ≤ 0.05, Tukey's adjustment. ns, not significant.

S1b Table. Impact of cover crop, fertilizer N rate, and cultivar on incidence of early blight and septoria leaf spot on tomato foliage in 2010 and 2011.

	# Infected leaves on 5 plants[a]				
	Early Blight			Septoria Leaf Spot	
	2010	2011		2011	
**Cover crop** [b]	Aug 8[c]	July 4[c]	July 20[c]	July 4[c]	July 20[d]
OSR	0.3 ns[e]	0.5 ns	0.7 ns	0.1 ns	5.0 ns
OSR+rye	0.0	0.2	1.2	0.0	3.6
No cover crop	0.1	0.2	0.6	0.0	3.6
Oat	0.3	0.6	1.8	0.0	3.4
Rye	0.0	0.2	1.6	0.0	3.7
**N fertilizer to tomatoes**					
Starter N only	0.2 ns	0.3 ns	1.1 ns	0.0 ns	4.2 ns
Full N	0.1	0.4	1.3	0.0	3.5
**Cultivar**					
Early	0.2 ns	0.4 ns	1.0 ns	0.0 ns	5.2 b
Late	0.1	0.3	1.4	0.0	2.7 a

[a] The number of infected leaves or fruit on five plants was recorded.
[b] Cover crop treatments were planted the autumn preceding tomato production. OSR, oilseed radish; rye, winter cereal rye.
[c] Data were not normal and could not be normalized using square root or log transformation.
[d] Data in these columns were normalized using a square root transformation; back transformed means are shown here.
[e] Different letters in each column indicates a statistical difference, $P \leq 0.05$, Tukey's adjustment. ns, not significant.

S1c Table. Impact of cover crop, fertilizer N rate, and cultivar on tomato foliar insect damage from Colorado potato beetle (CPB) and tomato hornworm (THW) in 2010 and 2011.

	CPB (2010 only)[a]				THW[a]	
	Incidence		Defoliation		# Defoliated Branches	
**Cover crop**[b]	June 21[c]	July 7[c]	June 21[c]	July 7[c]	July 26 2010[c]	Aug 11 2011[d]
OSR	0.1 ns[e]	0.1 ns	0.1 ns	0.3 ns	1.3 ns	3.7 ns
OSR+rye	0.3	0.2	3.4	0.8	2.6	3.5
No cover crop	0.1	0.1	0.1	0.1	0.6	3.2
Oat	0.1	0.1	0.4	0.4	3.4	4.6
Rye	0.1	0.1	0.1	0.1	2.8	3.7
**N fertilizer to tomatoes**						
Starter N only	0.2 ns	0.1 ns	1.6 ns	0.2 ns	2.2 ns	3.7 ns
Full N	0.1	0.2	0.1	0.5	2.0	3.8
**Cultivar**						
Early	0.2 ns	0.1 ns	1.5 ns	0.1 ns	1.8 ns	3.5 ns
Late	0.1	0.2	0.2	0.6	2.5	4.1

[a] For CPB, incidence is the number of leaves with feeding damage on five plants, and defoliation is the estimated amount of defoliation on the same five plants. For tomato hornworm (THW), the number of defoliated branches in the whole plot was recorded.

[b] Cover crop treatments were planted the autumn preceding tomato production. OSR, oilseed radish; rye, winter cereal rye.

[c] Data were not normal and could not be normalized using square root or log transformation.

[d] Data in these columns were normalized using a square root transformation; back transformed means are shown here.

[e] Different letters in each column indicates a statistical difference, $P \leq 0.05$, Tukey's adjustment. ns, not significant.

S1d Table. Impact of cover crop, fertilizer N rate, and cultivar on incidence of bacterial disease and stink bug damage on tomato fruit in 2010 and 2011.

	% bacterial spot or speck[a]			% stink bug damage[b]	
	2010	2011			
**Cover crop[c]**	Greens	Greens[d]	Reds[d]	2010[d]	2011[e]
OSR	22.7 ns [f]	6.6 ns	6.7 ns	4.9 ns	2.2 ns
OSR+rye	19.8	4.7	5.6	7.0	2.8
No cover crop	22.5	6.3	6.1	5.2	5.0
Oat	28.3	5.3	7.6	5.5	3.3
Rye	29.8	5.0	6.4	6.4	2.2
**N fertilizer to tomatoes**					
Starter N only	25.5 ns	4.5 ns	5.3 ns	6.5 ns	3.4 ns
Full N	23.8	6.7	7.7	5.1	2.8
**Cultivar**					
Early	5.7 a	4.1 ns	5.8 ns	6.3 ns	-
Late	43.5 b	7.2	7.1	5.3	-

[a] The percentage of tomatoes with spot or speck symptoms in a random sample of 50 fruit.
[b] The percentage of tomatoes with stink bug damage in a random sample of 50 red fruit.
[c] Cover crop treatments were planted the autumn preceding tomato production. OSR, oilseed radish; rye, winter cereal rye.
[d] Data in these columns was normalized using a square root transformation; back transformed means are shown here.
[e] Only early tomatoes were evaluated for stink bug damage in 2011.
[f] Different letters in each column indicates a statistical difference, $P \leq 0.05$, Tukey's adjustment. ns, not significant.

S1e Table. Impact of cover crop, fertilizer N rate, and cultivar on anthracnose incidence[a] and severity[b] on tomato fruit in 2010 and 2011.

	2010		2011[c]			
				Severity		
					Full N	
**Cover crop[d]**	Incidence (%)[c]	Severity	Incidence (%)	No N[c]	Early[c]	Late
OSR	6.6 ns [e]	4.7 ns	17.0 ns	9.2 ns	2.5 ns	24.7 ns
OSR+rye	8.3	5.3	17.4	6.9	3.6	23.5
No cover crop	9.7	7.4	19.8	16.4	2.5	12.7
Oat	7.1	4.6	17.0	12.2	2.1	14.0
Rye	7.3	5.0	14.0	7.2	4.4	10.3
**N fertilizer to tomatoes**						
Starter N only	7.5 ns	5.3 ns	18.7 ns	-	-	-
Full N	8.0	5.5	16.4	-	-	-
**Cultivar**						
Early	6.2 ns	4.2 ns	7.9 a	5.3 a	-	-
Late	9.5	6.6	27.2 b	16.5 b	-	-

[a] The percentage of tomatoes with anthracnose symptoms in sample of 50 red fruit reported for incidence.
[b] Tomatoes were sorted into classes 0 to 3, where 0, no symptoms; 1, one lesion; 2, two lesions; and 3, three or more lesions. A disease severity index (DSI) was calculated using the following equation: DSI = ∑[(# class 0 samples*)+(# class 1 samples*1)+(# class 2 samples*2)+(# class 3 samples*3)]/[(# classes – 1)*number samples]*100.
[c] Data in these columns were normalized using a square root transformation; back transformed means are shown here.
[d] Cover crop treatments were planted the autumn preceding tomato production. OSR, oilseed radish; rye, winter cereal rye.
[e] Different letters in each column indicates a statistical difference, $P \leq 0.05$, Tukey's adjustment. ns, not significant.
