# Supplementary material for: Winter cover crops on processing tomato yield, quality, pest pressure, nitrogen availability, and profit margins
Source: PLoS One. 2017 Jul 6;12(7):e0180500. doi: 10.1371/journal.pone.0180500 (PMC5500340; doi:10.1371/journal.pone.0180500)
Supplement: S2 Table — NTSS, natural tomato soluble solids; OSR, oilseed radish; rye, winter cereal rye. (PDF) [file pone.0180500.s005.pdf]

S2 Table. Impact of cover crop, N rate and cultivar on processing tomato quality in 2010 and 2011.

Treatment			
**Cover crop [CC][a]**	**Agtron colour**	**NTSS**	**pH**
**No cover crop**	18.2	4.24	4.34
**Oat**	19.4	4.23	4.33
**OSR**	18.6	4.26	4.34
**OSR + rye**	18.2	4.18	4.34
**Rye**	18.3	4.17	4.34
**SE**	0.452	0.077	0.007
**N rate [N]**			
**Starter N**	18.2	4.21	4.34
**Full N**	18.8	4.22	4.33
**SE**	0.322	0.067	0.004
**Cultivar [C]**			
**Early**	17.8	4.17	4.27
**Late**	19.2	4.26	4.40
**SE**	0.322	0.067	0.004
**Effect**	--------	P values	--------
**CC**	0.1814	0.6404	0.7748
**N**	0.1057	0.7752	0.2331
**C**	**0.0003**	0.0648	**<.0001**
**N*C**	0.8454	0.1957	0.1362
**CC*N**	0.8166	0.6094	0.5583
**CC*C**	0.5264	**0.0217**	0.5503
**CC*N*C**	0.9835	0.7767	0.8963

Cultivar by cover crop interaction for soluble solids

**Cultivar**	**Cover crop**	**NTSS**
Early	No cover crop	4.26
Early	OSR	4.33
Early	Oat	4.13
Early	Rye	4.13
Early	OSR + rye	4.02
Late	No cover crop	4.23
Late	OSR	4.20
Late	Oat	4.33
Late	Rye	4.21
Late	OSR + rye	4.33
	SE	0.0924

NTSS, natural tomato soluble solids; OSR, oilseed radish; rye, winter cereal rye.

[a]Cover crop treatments were planted the autumn preceding tomato production.
